# Population pharmacokinetics of trastuzumab emtansine (T-DM1), a HER2-targeted antibody–drug conjugate, in patients with HER2-positive metastatic breast cancer: clinical implications of the effect of covariates

**DOI:** 10.1007/s00280-014-2500-2

**Published:** 2014-06-18

**Authors:** Dan Lu, Sandhya Girish, Yuying Gao, Bei Wang, Joo-Hee Yi, Ellie Guardino, Meghna Samant, Melody Cobleigh, Mothaffar Rimawi, Pierfranco Conte, Jin Yan Jin

**Affiliations:** 1 Department of Clinical Pharmacology, Genentech, Inc., 1 DNA Way, South San Francisco, CA 94080 USA; 2 Drug Development Consulting Services, Quantitative Solutions, 845 Oak Grove Ave, Menlo Park, CA 94025 USA; 3 BioAnalytical Sciences, Genentech, Inc., 1 DNA Way, South San Francisco, CA 94080 USA; 4 Biostatistics, Genentech, Inc., 1 DNA Way, South San Francisco, CA 94080 USA; 5 Department of Internal Medicine, Rush University Medical Center, 1653 W. Congress Parkway, Chicago, IL 60612 USA; 6 Lester and Sue Smith Breast Center, Baylor College of Medicine, 6620 Main St., Houston, TX 77030 USA; 7 Department of Surgery, Oncology and Gastroenterology, University of Padua, Via Gattamelata 64, 35128 Padua, Italy

**Keywords:** Ado-trastuzumab emtansine, T-DM1, HER2, Pharmacokinetics, Metastatic breast cancer

## Abstract

**Purpose:**

Trastuzumab emtansine (T-DM1) is an antibody–drug conjugate comprising the humanized monoclonal antibody trastuzumab linked to DM1, a highly potent cytotoxic agent. A population pharmacokinetic (PK) analysis was performed to estimate typical values and interindividual variability of T-DM1 PK parameters and the effects of clinically relevant covariates.

**Methods:**

Serum samples were collected from 671 patients with human epidermal growth factor receptor 2-positive locally advanced or metastatic breast cancer (MBC) who received single-agent T-DM1 in five phase I to phase III studies. Nonlinear mixed-effects modeling with the first-order conditional estimation method was used.

**Results:**

A linear two-compartment model with first-order elimination from the central compartment described T-DM1 PKs in the clinical dose range. T-DM1 elimination clearance was 0.676 L/day, volume of distribution in the central compartment (*V*_c_) was 3.127 L, and terminal elimination half-life was 3.94 days. Age, race, region, and renal function did not influence T-DM1 PK. Given the low-to-moderate effect of all statistically significant covariates on T-DM1 exposure, none of these covariates is expected to result in a clinically meaningful change in T-DM1 exposure.

**Conclusions:**

T-DM1 PK properties are consistent and predictable in patients. A further refinement of dose based on baseline covariates other than body weight for the current 3.6 mg/kg regimen would not yield clinically meaningful reductions in interindividual PK variability in patients with MBC.

**Electronic supplementary material:**

The online version of this article (doi:10.1007/s00280-014-2500-2) contains supplementary material, which is available to authorized users.

## Introduction

Trastuzumab emtansine (T-DM1) is an antibody–drug conjugate (ADC) comprising the humanized monoclonal antibody (mAb) trastuzumab conjugated to the highly potent cytotoxic agent DM1. T-DM1 delivers DM1 specifically to human epidermal growth factor receptor 2 (HER2)-overexpressing tumor cells [1, 2]. Like trastuzumab, T-DM1 induces antibody-dependent cellular cytotoxicity, inhibits cell signaling through the phosphatidylinositol-3-kinase/AKT pathway, and inhibits HER2 shedding [2, 3]. Single-agent T-DM1 demonstrated superior efficacy in treating HER2-positive metastatic breast cancer (MBC) in the first-line and relapsed/refractory settings [4, 5] and was recently approved by the US Food and Drug Administration for the treatment of HER2-positive MBC [6].

The pharmacokinetics (PKs) of various analytes (T-DM1 conjugate, total trastuzumab, and DM1) after T-DM1 dosing were characterized in phase I–III studies by non-compartmental analysis (NCA) [5, 7]. Phase I data suggested that T-DM1 conjugate, the major analyte correlated with treatment efficacy and safety, exhibits faster clearance (CL) at doses ≤1.2 mg/kg and linear PKs for doses ≥2.4 mg/kg for the every 3-week (q3w) regimen [8]. In phase II and phase III studies with 3.6 mg/kg q3w, linear PK properties were confirmed, and no significant accumulation was observed, consistent with its terminal half-life of approximately 4 days [9–12]. For the weekly (qw) regimen, modest accumulation of T-DM1 conjugate was observed after the first three doses [13].

By targeting delivery, T-DM1 improves the therapeutic window of DM1. However, based on the maximum tolerated dose (MTD) identified for both q3w and qw regimens, T-DM1 has a relatively narrow therapeutic window compared with typical mAbs [7, 8, 13]. Therefore, it is important to assess the effects of demographic and pathophysiologic covariates on the PKs of T-DM1 to determine whether dose adjustments are needed. To estimate typical PK parameter values and interindividual variability (IIV) for T-DM1 conjugate, an interim population PK (PopPK) model was developed using PK data from 273 patients in one phase I (TDM3569g) and two phase II studies (TDM4258g and TDM4374g) [14]. To support the labeling statement on the PK properties of T-DM1 and dosing recommendation, an updated PopPK model including data from an additional 400 patients from one phase II TDM4450g trial and one phase III registrational trial (EMILIA) is reported here. Relative to the previous report [14] and to better characterize the effects of covariates on T-DM1 PK in patients with breast cancer, a more comprehensive spectrum of covariates with a wider range of values was evaluated. The updated PopPK model was also externally validated by another phase II study (TDM4688g). The model reported here will be of great value for the current clinical application of T-DM1 in patients with breast cancer.

## Materials and methods

### Patients, PK serum sampling, and bioanalysis

After the administration of single-agent T-DM1, PK data were collected from patients participating in the TDM3569g, TDM4258g, TDM4374g, TDM4450g, TDM4370g (EMILIA), and TDM4688g trials [4, 5, 8–10, 13, 15] (Supplemental Table 1). All study designs were approved by independent ethics committees and conducted in accordance with the Declaration of Helsinki; all patients provided written informed consent [4, 5, 8–10, 13, 15].

T-DM1 serum samples were analyzed by the Bioanalytical Sciences Department at Genentech, Inc., (South San Francisco, CA) or by PPD (Richmond, VA) using a validated indirect sandwich enzyme-linked immunosorbent assay (Genentech, Inc., data on file). The conjugate assay quantified all conjugated trastuzumab containing ≥1 covalently bound DM1 molecule, while excluding unconjugated trastuzumab. The minimum quantifiable concentration (MQC) of the assay ranged from 0.04 to 0.06 µg/mL [16]. Observations below the MQC were omitted from the analysis.

### Establishment and validation of the PopPK final model

PopPK analysis was performed using nonlinear mixed-effects modeling with first-order conditional estimation with interaction method. Model estimation and evaluation were implemented with NONMEM 7 (version 7.1.2; ICON Development Solutions, Ellicott City, MD) with Intel Fortran Compiler (version 10.1.021; Intel, Santa Clara, CA), PerlSpeaksNONMEM (version 3.2.12; Uppsala University, Uppsala, Sweden), and S-PLUS 6.2 (TIBCO Software Inc., Palo Alto, CA).

A base model was first established, with all covariates likely to impact T-DM1 conjugate exposure (Supplemental Table 2) explored for a possible correlation with key T-DM1 post hoc PK parameters. The clinically relevant covariates tested included those related to demographics, renal function, disease severity, and treatment history. The final model was identified by testing covariates using stepwise forward addition followed by backward deletion. A change in minimum objective function (MOF) at the *P* < 0.01 level of significance (log-likelihood ratio test) was used for the forward addition step, and *P* < 0.001 was used for the backward deletion step to retain the covariates in the final model.

Internal validation was performed, including goodness-of-fit diagnostics, visual predictive check (VPC) plots [17, 18], numerical predictive check (NPC) [18], bootstrap [19], and shrinkage [20] assessments. Compared with internal validation, external validation is more rigorous because the model’s predictability is evaluated against a new dataset [21–23]. PK data from the phase II study TDM4688g were used for external validation (*N* = 51) [15]. Predicted T-DM1 serum concentrations for validation patients were obtained using post hoc Bayesian forecasting by fixing the parameters in the structural and variance models to the final estimates. Population-predicted serum T-DM1 concentrations (PRED) were compared with observed T-DM1 concentrations (DV). Prediction errors (*P*_e_) were calculated as (Eq. 1):

1 $$ P_{\text{e}} = \frac{{\left( {{\text{PRED}} - {\text{DV}}} \right)}}{\text{DV}} \times 100\;\% $$

Bias (mean prediction error [MPE]) was then calculated (Eq. 2):

2 $$ {\text{MPE}} = \frac{{\sum {P_{\text{e}} } }}{n} $$

where *n* denotes the number of observations.

The predicted clearance (CL_POP,*i*_) and central volume (*V*_cPOP,*i*_) for each patient (per individual covariate values) were obtained and compared with the maximum a posteriori probability (MAP) Bayesian estimates of clearance (CL_Bayesian,*i*_) and central volume (*V*_cBayesian,*i*_) for each validation patient based on the available concentration measurements and final PopPK parameter estimates. Prediction errors were calculated for each individual patient (*P*_ei_) and expressed as a percentage of the MAP Bayesian estimate (Eq. 3):

3 $$ \begin{gathered} {\text{CL}}\_P_{\text{ei}} = \frac{{{\text{CL}}_{{{\text{POP}},i}} - {\text{CL}}_{{{\text{Bayesian}},i}} }}{{{\text{CL}}_{{{\text{Bayesian}},i}} }} \times 100\;\% \hfill \\ V_{\text{c}} \_P_{\text{ei}} = \frac{{V_{{{\text{c}}\;{\text{POP}},i}} - V_{{{\text{c}}{\text{Bayesian}},i}} }}{{V_{{{\text{c}}{\text{Bayesian}},i}} }} \times 100\;\% \hfill \\ \end{gathered} $$

The bias of prediction was assessed by MPE (Eq. 4):

4 $$ {\text{MPE}} = \frac{{\sum {P_{\text{ei}} } }}{N} $$

where *N* denotes the number of patients.

Based on the final model, the effect of extreme values of each statistically significant covariate (5th and 95th percentiles) on T-DM1 PK parameters (CL and *V*_c_) was evaluated. To assess whether T-DM1 PKs differed in various populations, individual Bayesian post hoc CL and *V*_c_ were normalized by statistically significant covariates in the final model and compared among clinically relevant populations (e.g., those defined by age, race, region, renal function, disease severity, and treatment history).

### Sensitivity analyses

Sensitivity analyses were performed to examine the influence of statistically significant covariates on the expected steady-state exposure of T-DM1 conjugate, including steady-state area under the concentration versus time curve (AUC), maximum concentration (*C*_max_), and trough concentration (*C*_trough_). The simulated exposure of patients with extreme covariate values (5th and 95th percentiles) was compared with a typical patient with median covariate values for each of the statistically significant covariates in the final model.

### Model applications: exposure comparison among various populations

To compare the expected T-DM1 conjugate steady-state exposure in populations defined by age, race, region, or renal function, individual T-DM1 exposures (steady-state AUC, *C*_max_, *C*_trough_) were simulated using Bayesian post hoc PK parameters for each patient for multiple doses of 3.6 mg/kg q3w. This simulation accounts for potential correlations among covariates.

## Results

### PopPK analysis datasets

The development dataset for the final model included 9,934 T-DM1 conjugate serum concentration–time data points from 671 participants in the TDM3569g, TDM4258g, TDM4374g, TDM4450g, and TDM4370g (EMILIA) trials. Of these patients, 643 (95.8 %) received T-DM1 q3w and 28 (4.2 %) received T-DM1 qw. The external validation dataset contained 505 concentration–time data points from 51 participants administered T-DM1 q3w in TDM4688g. Baseline demographic and clinical characteristics of patients included in the PopPK analysis are shown in Supplemental Table 3. In total, 7.27 % of all data points were below the MQC and thus excluded from the analysis.

### Final PopPK model

A linear two-compartment model with first-order elimination from the central compartment best described T-DM1 conjugate serum concentration–time data (Supplemental Fig. 1). The final PopPK model parameter–covariate relations were as follows (Eq. 5):

5 $$ \begin{aligned} {\text{CL}}_{i} & = \exp \left( {\theta_{1} + \theta_{6} \cdot { \log }\left( {\frac{\text{weight}}{70}} \right) + \theta_{7} \cdot { \log }\left( {\frac{\text{ECD}}{25}} \right) + \theta_{8} \cdot { \log }\left( {\frac{\text{ALBU}}{41}} \right)} \right. \\ & \quad \left. { + \;\theta_{9} \cdot { \log }\left( {\frac{\text{TMBD}}{9}} \right) + \theta_{10} \cdot {\text{TBL}} + \theta_{11} \cdot { \log }\left( {\frac{\text{AST}}{27}} \right) + \eta_{_{\text{CL}}} } \right) \\ V_{{c_{i} }} & = \exp \left( {\theta_{2} + \theta_{5} \cdot { \log }\left( {\frac{\text{weight}}{70}} \right) +  \eta_{_{V_{\text{c}} }} } \right) \\ \end{aligned} $$

where CL_*i*_, individual patient elimination clearance; *η*_CL_, IIV of CL; $\eta_{_{V_{\text{c}}}}$, IIV of *V*_c_; *θ*_1_, typical value of CL; *θ*_2_, typical value of *V*_c_; *θ*_5_, influence of body weight on *V*_c_; *θ*_6_, influence of body weight on CL; *θ*_7_, influence of baseline serum HER2 shed extracellular domain concentration (ECD) on CL; *θ*_8_, influence of serum albumin concentration (ALBU) on CL; *θ*_9_, influence of the baseline sum of the longest dimension of target lesions (TMBD) on CL; *θ*_10_, influence of baseline trastuzumab concentration (TBL) on CL; *θ*_11_, influence of serum aspartate aminotransferase concentration (AST) on CL; *V*_ci_, individual patient volume of distribution in the central compartment.

Based on the identified statistically significant covariates on CL and *V*_c_, patients with higher body weight, ECD, TMBD, or AST, or those with lower ALBU or TBL, had higher CL; patients with higher baseline body weight had higher *V*_c_. In the PopPK model, the estimated typical CL and *V*_c_ for T-DM1 were 0.676 L/day and 3.127 L, respectively (Table 1). The typical value for the terminal elimination half-life was 3.94 days, suggesting that T-DM1 conjugate does not accumulate after repeated q3w dosing, and steady state is reached during the first cycle. The IIV estimated for T-DM1 CL and *V*_c_ from the base model without covariates was 25.6 and 17.5 %, respectively, and was further reduced in the final model (after incorporating covariate effects) to 19.1 and 11.7 %, respectively; all covariates together explained 44.4 and 55.8 % of the IIV in CL and *V*_c_ in the base model, respectively.

**Table 1:** Typical and 95 % CIs for PopPK parameter estimates from the final model

Parameter	Parameter description	Final PopPK model point estimates (95 % CI)
exp(*θ*_1_)*24	CL (L/day)	0.676 (0.661–0.691)
*θ*_6_	Influence of weight on CL	0.49 (0.41–0.57)
*θ*_7_	Influence of ECD on CL	0.035 (0.021–0.05)
*θ*_8_	Influence of ALBU on CL	–0.423 (–0.553 to –0.293)
*θ*_9_	Influence of TMBD on CL	0.052 (0.033–0.071)
*θ*_10_	Influence of TBL on CL	−0.002 (−0.002 to −0.001)
*θ*_11_	Influence of AST on CL	0.071 (0.036–0.106)
exp(*θ*_2_)	*V*_c_ (L)	3.127 (3.08–3.174)
*θ*_5_	Influence of weight on *V*_c_	0.596 (0.526–0.666)
exp(*θ*_3_)*24	Distribution CL (Q; L/day)	1.534 (1.286–1.83)
exp(*θ*_4_)	*V*_p_ (L)	0.66 (0.58–0.752)
Interindividual variability (%)	CL	19.11 (17.58–20.52)
	*V*_c_	11.66 (10.18–12.975)
	*Q*	180.8 (165.8–194.7)
	*V*_p_	74.50 (62.73–84.65)
$\omega_{{{\text{CL}},V_{\text{c}} }}^{2}$	Covariance between CL and *V*_c_	0.011 (0.008–0.015)
Σ	Residual variability (% CV)	31.56 (31.07–32.04)

*%CV* the mean percentage coefficient of variation, *ALBU* serum albumin concentration, *AST* serum aspartate aminotransferase concentration, *CI* confidence interval, *CL* elimination clearance, *ECD* baseline serum human epidermal growth factor receptor 2 shed extracellular domain concentration, *PopPK* population pharmacokinetic, *Q* distribution clearance, *TBL* baseline trastuzumab concentration, *TMBD* baseline sum of longest dimension of target lesions, *V*_c_ volume of distribution in the central compartment, *V*_p_ volume of distribution in the peripheral compartment

Goodness-of-fit plots showed good agreement between predicted and observed concentrations of T-DM1 (Supplemental Fig. 2), with no apparent bias in residual plots over time or across population-predicted concentrations (Supplemental Fig. 3). VPC plots (Supplemental Fig. 4A) and NPC (data not shown) showed that the final PopPK model could adequately reproduce the central tendency and variability of the T-DM1 conjugate serum concentrations across all studies for the labeled regimen, 3.6 mg/kg q3w.

Bootstrapping of 1,000 datasets resulted in median parameter estimates and 95 % confidence intervals (CIs) similar to the estimates from the original dataset (data not shown), indicating that the final PopPK model provided good precision for parameter estimation. The ε-shrinkage was 6.1 %, and η-shrinkage [20] for CL was 15.6 %; therefore, the Bayesian estimates for CL were robust enough to estimate the relationship between CL and related covariates. The η-shrinkage for *V*_c_, distribution clearance (*Q*), and volume of distribution in the peripheral compartment (*V*_p_) were 38.5, 49.7, and 36.1 %, respectively.

The external validation dataset was well predicted by the final model based on goodness-of-fit plots (data not shown), VPC (Supplemental Fig. 4B), and NPC (data not shown). No bias was observed over time or across PRED. The final PopPK model accurately predicted T-DM1 conjugate concentrations, individual CL and *V*_c_ in the validation patients with minimal bias (represented by MPE values) of 2.70 % (95 % CI −9.87 to 4.48), 1.97 % (95 % CI −7.17 to 3.23), and −2.20 % (95 % CI −4.25 to –0.15), respectively. None significantly differed from zero at the *P* > 0.01 level of significance.

### Impact of covariates on PK parameters

The effect of extreme values (5th and 95th percentiles for 671 patients) of statistically significant covariates on T-DM1 CL and *V*_c_ was assessed. Despite statistical significance, the impact of their variation for a single covariate on key T-DM1 PK parameters was low: <20 % for CL and <25 % for *V*_c_ (Table 2).

**Table 2:** Effect of covariates on T-DM1 PK parameters

PK parameters and baseline covariates	Baseline covariate value	Estimate	Percent change from typical
Typical CL (L/day)^a^		**0.676**	
Body weight (kg)			
5th percentile	49	0.567	–16.04
95th percentile	98	0.797	17.92
ECD (ng/mL)			
5th percentile	8.5	0.650	–3.747
95th percentile	332	0.741	9.588
ALBU (g/L)			
5th percentile	33	0.741	9.617
95th percentile	48	0.632	–6.450
TMBD (cm)			
5th percentile	1.5	0.616	–8.831
95th percentile	30.3	0.719	6.464
TBL (µg/mL)			
5th percentile	0	0.676	0.000
95th percentile	54	0.615	–9.017
AST (IU/L)			
5th percentile	15.3	0.649	–3.936
95th percentile	64	0.718	6.292
Typical *V*_c_ (*L*) for a 70-kg patient		**3.127**	
Body weight (kg)			
5th percentile	49	2.523	–23.69
95th percentile	98	3.821	18.17

*ALBU* serum albumin concentration, *AST* serum aspartate aminotransferase concentration, *CL* elimination clearance, *ECD* baseline serum human epidermal growth factor receptor 2 shed extracellular domain concentration, *PK* pharmacokinetic, *TBL* baseline trastuzumab concentration, *T*-*DM1* trastuzumab emtansine, *TMBD* baseline sum of longest dimension of target lesions, *V*_c_ volume of distribution in the central compartment

^a^A 70-kg patient with ECD of 25 ng/mL, ALBU of 41 g/L, TMBD of 9 cm, TBL of 0 µg/mL, and AST of 27 U/L

**Fig. 1:**
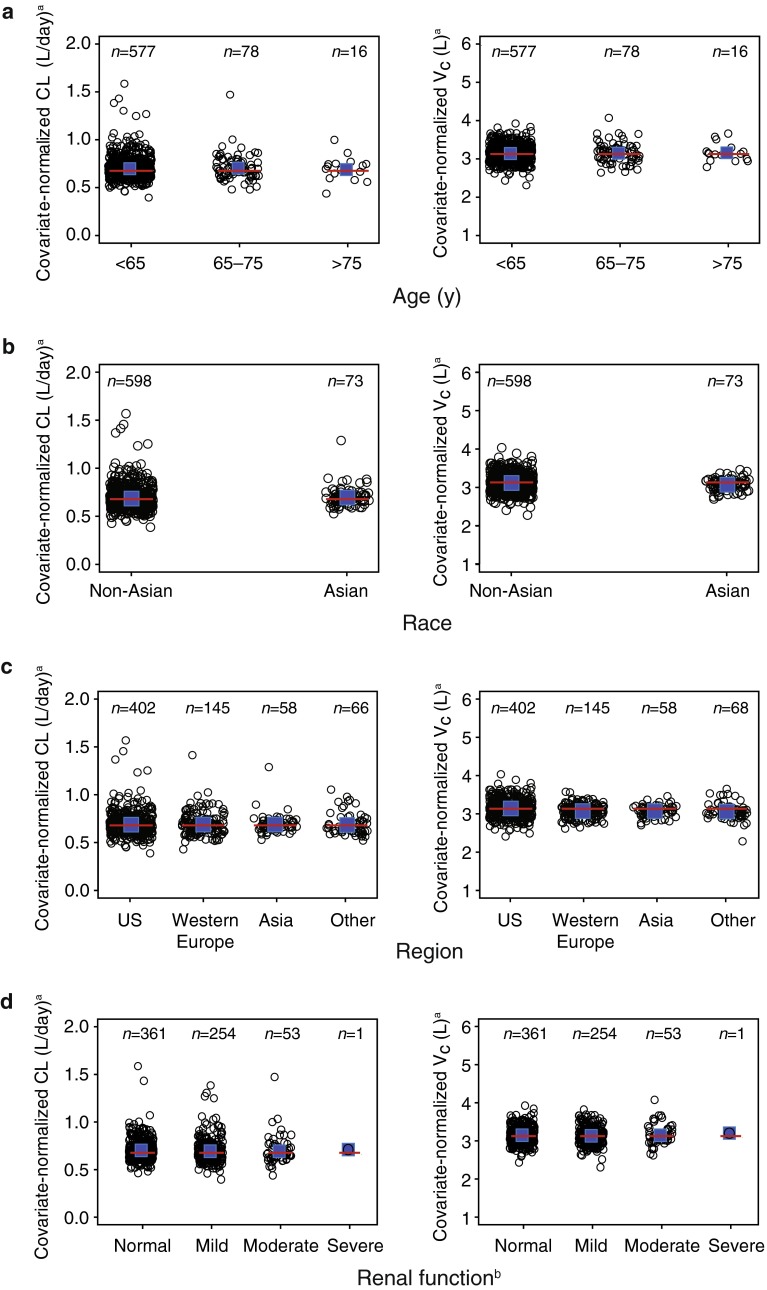
Comparison of T-DM1 PK parameters^a^ for patients by **a** age, **b** race, **c** region, and **d** renal function^b^. ^a^Normalized for body weight of 70 kg, ECD of 25 ng/mL, ALBU of 41 g/L, TMBD of 9 cm, TBL of 0 μg/mL, and AST of 27 U/L. ^b^Normal: CrCL ≥ 90 mL/min; mild: CrCL = 60–89 mL/min; moderate: CrCL = 30–59 mL/min; severe: CrCL = 15–29 mL/min. *Circles* indicate individual CL or *V*_c_ estimates. The *red lines* indicate typical population-predicted covariate relationships, and the *blue squares* are the means of individual estimates. *ALBU* serum albumin concentration, *AST* serum aspartate aminotransferase concentration, *CL* elimination clearance, *CrCL* baseline creatinine clearance, *ECD* baseline serum human epidermal growth factor receptor 2 shed extracellular domain concentration, *PK* pharmacokinetic, *TBL* baseline trastuzumab concentration, *T*-*DM1* trastuzumab emtansine, *TMBD* baseline sum of the longest dimension of the target lesion, *US* United States, *V*_c_ volume of distribution in the central compartment, *VPC* visual predictive check

Age, race, and calculated baseline creatinine clearance (CrCL) using the Cockcroft-Gault formula [24, 25] were not statistically significant covariates for PK parameters. Patients grouped by age, race, geographic region, and renal function (normal vs. mild impairment vs. moderate impairment based on CrCL [25]) had similar covariate-normalized CL or *V*_c_ (Fig. 1). The lower Bayesian post hoc CL and *V*_c_ estimates observed for Asian patients were likely due to the covariate effect of body weight on CL and *V*_c_, as Asian patients had a slightly lower body weight versus non-Asian patients (mean body weight, 60.5 versus. 71.6 kg ).

**Fig. 2:**
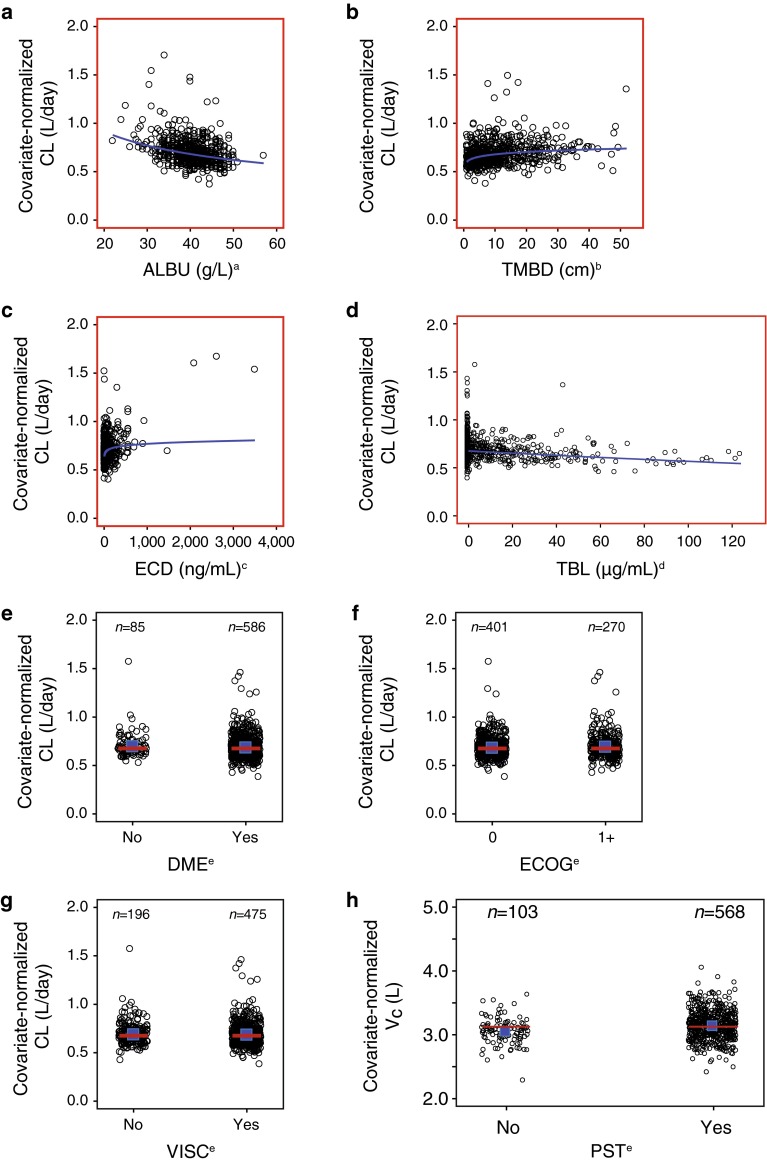
T-DM1 PK parameter–covariate relationships for baseline disease severity and prior treatment history, **a** ALBU, **b** TMBD, **c** ECD, **d** TBL, **e** DME, **f** ECOG, **g** VISC, **h** PST. ^a^Normalized for body weight of 70 kg, TBL of 0 μg/mL, ECD of 25 ng/mL, AST of 27 U/L, and TMBD of 9 cm. ^b^Normalized for body weight of 70 kg, ALBU of 41 g/L, TBL of 0 μg/mL, ECD of 25 ng/mL, and AST of 27 U/L. ^c^Normalized for body weight of 70 kg, ALBU of 41 g/L, TBL of 0 μg/mL, AST of 27 U/L, and TMBD of 9 cm. ^d^Normalized for body weight of 70 kg, ALBU of 41 g/L, ECD of 25 ng/mL, AST of 27 U/L, and TMBD of 9 cm. ^e^Normalized for body weight of 70 kg, ALBU of 41 g/L, TBL of 0 μg/mL, ECD of 25 ng/mL, AST of 27 U/L, and TMBD of 9 cm. Points indicate individual parameter estimates. In **a**–**b** *blue lines* indicate a typical (population) predicted covariate relationship. The *red boxes* represent a statistically significant PK parameter–covariate relationship. In **e**–**g** *red lines* indicate a typical (population) predicted covariate relationship. The *blue squares* represent the means of individual estimates. *ALBU* serum albumin concentration, *AST* serum aspartate aminotransferase concentration, *CL* elimination clearance, *DME* disease measurability, *ECD* baseline serum human epidermal growth factor receptor 2 shed extracellular domain concentration, *ECOG* baseline Eastern Cooperative Oncology Group performance status score, *PK* pharmacokinetic, *PST* prior systemic therapy in the locally advanced/metastatic setting, *TBL* trastuzumab baseline concentration, *T*-*DM1* trastuzumab emtansine, *TMBD* baseline sum of the longest dimension of target lesions, *VISC* visceral disease

ALBU, TMBD, and ECD were disease severity-related baseline covariates identified as being statistically significant for T-DM1 CL in the final PopPK model (Fig. 2). Patients with lower ALBU or higher TMBD or ECD tended to have higher CL; however, the extreme values of a single covariate on CL resulted in a <10 % change from a typical patient (Table 2). Other covariates related to disease severity (e.g., disease measurability, visceral disease, and Eastern Cooperative Oncology Group performance status) did not affect CL (Fig. 2) or *V*_c_ (data not shown).

Among covariates related to treatment history, TBL was identified as a statistically significant covariate for T-DM1 CL but not for *V*_c_. The extreme values of a single covariate on CL resulted in a <10 % change from a typical patient (see Table 2). Whether patients received prior systemic therapy in the locally advanced or metastatic settings did not appear to affect CL (Fig. 2) or *V*_c_ (data not shown).

### Sensitivity analyses

**Fig. 3:**
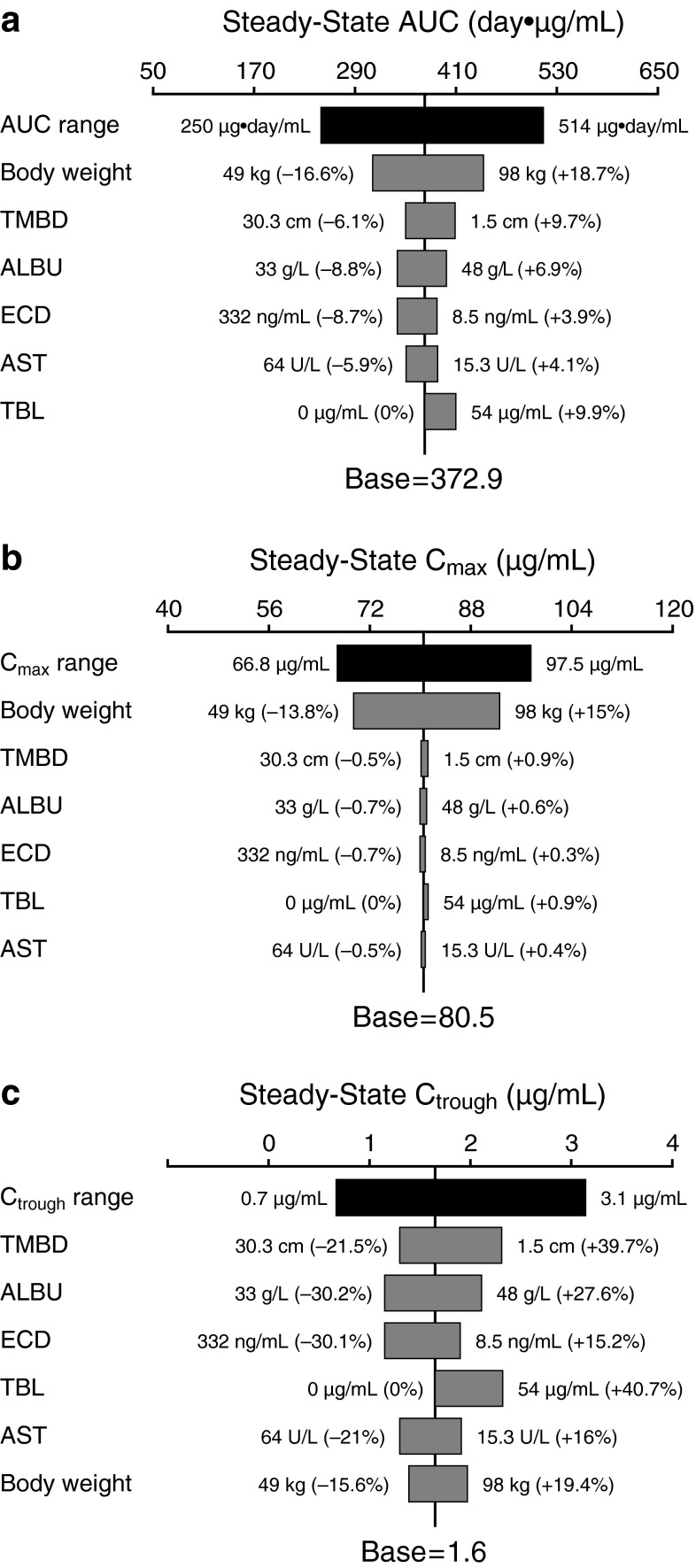
Sensitivity plot comparing the effect of covariates on T-DM1 steady-state exposure after repeated dosing (3.6 mg/kg q3w): **a** AUC, **b** *C*_max_, and **c** *C*_trough_. *Black vertical line* indicates the base predicted steady-state exposure of T-DM1 in a typical patient with a body weight of 70 kg, ECD of 25 ng/mL, ALBU of 41 g/L, TMBD of 9 cm, TBL of 0 μg/mL, and AST of 27 U/L. The *blue shaded bar* represents the 5th to 95th percentile. *Values* in parentheses indicate percent change of exposure from base. The *upper* and *lower* values for each covariate capture 90 % of the plausible range in the population. The length of each bar represents the potential effect of that particular covariate on T-DM1 exposure at steady state. *ALBU* serum albumin concentration, *AST* serum aspartate aminotransferase concentration, *AUC* area under the serum concentration versus time curve, *C*_max_ maximum concentration, *C*_trough_ trough concentration, *ECD* baseline serum human epidermal growth factor receptor 2 shed extracellular domain concentration, *q3w* every 3 weeks, *TBL* baseline trastuzumab concentration, *T*-*DM1* trastuzumab emtansine, *TMBD* baseline sum of the longest dimension of the target lesion

Sensitivity analyses (Fig. 3) suggested that the magnitude of effect of all statistically significant covariates on T-DM1 conjugate steady-state AUC (<19 %) and *C*_max_ (<15 %) was low and moderate on T-DM1 conjugate *C*_trough_ (<41 %). Of note, *C*_trough_ is associated with greater variability than AUC and *C*_max_. Baseline body weight was the covariate with the greatest effect on T-DM1 steady-state AUC and *C*_max_ (see Fig. 3). Given the low IIV of T-DM1 key PK parameters (CL and *V*_c_) and the low-to-moderate effect of all statistically significant covariates on T-DM1 exposure (*C*_max_, *C*_trough_, AUC), no covariate is expected to have clinically meaningful effects on T-DM1 exposure.

### Model applications: exposure comparison among various populations

All exposure parameters were similar across age groups (<65, 65–75, >75 years) (Supplemental Table 4). Thus, dose adjustment in elderly patients is not justified.

Asian patients and patients enrolled in Asia had a <7 % lower mean AUC with largely overlapping intervals of the 5th to 95th percentile (Supplemental Table 4). However, this difference is likely due to body weight rather than to race or region. Asian patients had an approximately 16 % lower body weight (60.5 kg) versus non-Asian patients (71.6 kg) and received a lower amount of T-DM1 under body weight-based dosing. Thus, no dose adjustment based on race or region is considered necessary.

Patients with mild or moderate renal impairment had a <11 % lower mean AUC value with largely overlapping intervals of the 5th to 95th percentile (Supplemental Table 4). CrCL, as calculated by the Cockcroft-Gault formula [24, 25], is correlated with body weight. Due to their lower body weight, patients with mild or moderate renal impairment received lower amounts of T-DM1 under body weight-based dosing versus patients with normal renal function. As exposure differences are not caused directly by renal function, dose adjustment based on renal function is not necessary. However, because of the limited numbers of patients, no conclusions can be drawn regarding the effects of severe renal impairment (*n* = 1) or end-stage renal disease (*n* = 0) on T-DM1 exposure.

## Discussion

Results from this PopPK analysis informed the prescribing information for T-DM1 in patients with HER2-positive MBC, specifically its PK properties and dose justification based on the impact of weight, age, race, region, renal function, treatment history, and disease and health status on PKs. The model can reliably predict T-DM1 exposure and IIV irrespective of prior trastuzumab treatment.

Linear PKs were observed in patients receiving clinically relevant T-DM1 doses (2.4–4.8 mg/kg q3w). T-DM1 CL appeared to be faster in the five patients who received T-DM1 doses of ≤1.2 mg/kg q3w, likely due to target-mediated disposition at low doses. However, the PopPK model parameters associated with nonlinear elimination were not estimated with good precision, possibly because of the limited amount of data available for these lower doses of T-DM1. Sensitivity analyses indicated that the inclusion/exclusion of PK data from these five patients did not impact PK parameter estimation.

The T-DM1 CL (0.676 L/day) is approximately three times faster than that of unconjugated trastuzumab (approximately 0.2 L/day) [26]. The elimination half-life of T-DM1 (approximately 4 days) is shorter than that for a typical immunoglobulin G1 antibody (2–3 weeks) [27]. These data support multiple mechanisms of T-DM1 CL. T-DM1 undergoes both target-mediated (HER2) and non-specific (partly Fc-mediated) proteolytic degradation, similar to that of mAbs [28]. Moreover, DM1 deconjugation from T-DM1 may contribute to T-DM1 CL, which may partially explain the faster CL and shorter half-life of T-DM1 [29]. The central volume of distribution of T-DM1 for a 70-kg patient is 3.127 L, which is similar to that of unconjugated trastuzumab (~2.95 L) ([26]), other mAbs (~2.4–5.5 L) ([27]), and ADCs such as brentuximab vedotin (4.16 L) [30]. The peripheral volume of distribution of T-DM1 is relatively small (0.66 L) and is lower than that of brentuximab vedotin (8.06 L) [30]. Overall, the PK properties of T-DM1 are more similar to a mAb than to a small molecule drug, with relatively slow CL and a small volume of distribution that is mostly confined to plasma.

The linker used in ADCs may affect CL by impacting the rate of deconjugation of the cytotoxic drug component. Several linkers have been used in various compounds in clinical development. For example, T-DM1 contains a non-cleavable thioether linker (4-(*N*-malemidomethyl) cyclohexane-1-carboxylate [MCC]); brentuximab vedotin contains a peptide-based linker (maleimidocaproyl-valine-citruline-p-aminobenzyl-oxycarbonyl [MC-vc-PAB]); inotuzumab ozogamicin and gemtuzumab ozogamicin contain acid-labile linkers (4-(4-acetylphenoxy)butanoic acid [AcBut]); and AVE9633 and SVR3419 contain disulfide linkers (*N*-succinimidyl-3-(2′-pyridyldithio) butyrate [SPDB]). T-DM1, with its MCC linker, has a CL rate (~0.7 L/day) similar to ADCs containing SPDB linkers (AVE9633 200–260 mg/m^2^, ~0.9–1.1 L/day [31, 32]; SAR3419, ~0.6 L/day [33]). However, higher CL values have been observed for ADCs containing MC-vc-PAB linkers (brentuximab vedotin 1.8 mg/kg, ~1.5–1.8 L/day [30, 32, 34]) or AcBut linkers (inotuzumab ozogamicin 1.8 mg/m^2^, ~2.2–3.8 L/day [32, 35, 36]; gemtuzumab ozogamicin 0.23 mg/kg, ~3.2 L/day [32, 37]). The lower CL of T-DM1 at clinically relevant doses (i.e., when target-mediated CL is largely saturated) might be attributable to its non-cleavable linker, which results in less rapid deconjugation of the cytotoxic component.

The relatively small IIV of T-DM1 CL and *V*_c_ (~19 and 12 %, respectively, Table 1) is consistent with the expected IIV of mAbs (~30–40 %) [27]. The IIV estimated for Q and V_p_ was relatively high in the final PopPK model, likely because of the limited data in the terminal phase of the phase II and phase III trials. A relatively large η-shrinkage of >30 % for *V*_c_, *Q*, and *V*_p_ was observed in the final model, suggesting that PK data may not sufficiently characterize the IIV for these parameters. More intensive concentration–time data may improve the accuracy of the IIV estimation, especially for *Q* and *V*_p_, which had relatively high η-shrinkage in both the base (data not shown) and final models.

The body weight-based regimen of 3.6 mg/kg was established as the MTD for T-DM1 in phase I testing [8]. Per a theoretical simulation comparing flat versus body weight-based dosing [38], flat dosing would not be expected to reduce IIV, given that body weight impacts T-DM1 CL and *V*_c_ with exponential function values of 0.5 and 0.6, respectively (Table 1, parameters *θ*_6_ and *θ*_5_). Sensitivity analyses suggested that the magnitude of the impact of body weight on T-DM1 PK parameters (CL, *V*_c_) and exposure (AUC, *C*_max_, *C*_trough_) is relatively small (<25 %) (Table 2; Fig. 3).

T-DM1 conjugate was cleared more slowly in patients with lower body weight (Table 2); however, conjugate exposure was lower in lighter patients due to body weight-based dosing (Fig. 3). Of the 671 patients included in this analysis, 68 (10.1 %) weighed ≥90 kg and thus received a greater amount of T-DM1 versus the overall population. These patients had higher mean exposure (21.0 % higher for AUC, 18.4 % higher for *C*_max_, 19.1 % higher for *C*_trough_, with largely overlapping intervals of 5th to 95th percentiles), despite faster CL and larger *V*_c_. Furthermore, based on the exposure–response analysis of T-DM1 3.6 mg/kg q3w [7, 12, 39, 40], the variability in T-DM1 AUC and *C*_max_ is not expected to have a clinically meaningful impact on overall safety; thus, the current body weight-based regimen remains appropriate, with no further dose adjustment recommended for heavier patients.

The covariates of age, race, and geographic region were not significant, suggesting that no further dose adjustment based on these covariates is necessary. Although Asian patients and patients from Asia have lower mean exposures, these differences are likely due to lower body weight. Based on the exposure–response analysis of T-DM1 3.6 mg/kg q3w [7, 12, 39, 40], this exposure variability is not expected to have a clinically meaningful impact on safety.

Compared with the earlier PopPK model that included only phase I and phase II data [14], two additional covariates (ECD and TBL) were identified as significantly impacting T-DM1 CL in the revised updated PopPK model. This may be a consequence of the increased number of patients in the second- and third-line treatment settings, patients who introduced a larger dynamic range for these two covariates. However, the effects of ECD and TBL were not considered clinically meaningful because of the small-to-moderate magnitude of effect on AUC, *C*_max_, and *C*_trough_. Mechanistically, ECD and TMBD are highly correlated with total HER2 antigen concentration, and their correlation with T-DM1 CL suggests a potential mechanism of HER2 target-mediated CL of T-DM1. These findings are similar to those from a PopPK analysis of trastuzumab, where ECD was also found to be a statistically significant covariate for trastuzumab CL [26]. While high baseline TBL resulting from prior trastuzumab treatment may increase total trastuzumab exposure and compete with T-DM1 for the HER2-mediated CL pathway, the effects of this covariate on exposure do not appear to translate into clinically relevant differences in safety [12, 39, 40].

Although TMBD (and potentially overall disease severity) for most patients decreased over time and after repeated T-DM1 infusions (because of shrinkage of target lesions following treatment), T-DM1 PKs do not appear to change over time. Model diagnostic plots did not suggest the existence of apparent bias of residual versus time (Supplemental Fig. 3), further indicating that inclusion of time-varying covariates for this patient population may not be necessary. Based on NCA, similar PK parameters were observed in cycle 1 and after several doses of T-DM1 (data not shown), suggesting that the PKs of T-DM1 are not largely affected by tumor size or changes in disease severity after treatment; this may be due to the relatively small magnitude of effect of TMBD on T-DM1 CL and exposure.

Given the high molecular weight of T-DM1, renal function is unlikely to impact CL. Based on preclinical studies, T-DM1 is mainly eliminated through bile after conversion to DM1-containing catabolites, with minimal (<5 %) renal elimination [41]. T-DM1 PK parameters (CL, *V*_c_) after normalization of other covariates were similar in patients with varying degrees of renal function (normal, mild impairment, or moderate impairment [per calculated CrCL]). Thus, renal impairment is not expected to impact T-DM1 PKs.

Some covariates related to hepatic function were tested in this analysis, with ALBU and AST identified as being statistically significant. A dedicated phase I study (BO25499) of T-DM1 in patients with MBC and normal or mild/moderate hepatic impairment is ongoing; PKs and safety results will be reported separately. PKs in patients with early-stage breast cancer characterized by lower baseline tumor burden and non-metastatic disease status are also being evaluated.

## Conclusion

The T-DM1 PK properties are consistent and predictable in patients with HER2-positive MBC. Age, race, region, and renal function did not influence T-DM1 PKs. Given the low IIV of T-DM1 key PK parameters (CL and *V*_c_) and the low-to-moderate effect of statistically significant covariates on T-DM1 exposure, a further refinement of dose based on baseline covariates other than body weight for the current 3.6 mg/kg regimen would not yield clinically meaningful reductions in interindividual PK variability in this patient population.

## Electronic supplementary material

Below is the link to the electronic supplementary material.

Supplementary material 1 (DOC 450 kb)
