# Supplementary material for: Population pharmacokinetics of trastuzumab emtansine (T-DM1), a HER2-targeted antibody–drug conjugate, in patients with HER2-positive metastatic breast cancer: clinical implications of the effect of covariates
Source: Cancer Chemother Pharmacol. 2014 Jun 18;74(2):399–410. doi: 10.1007/s00280-014-2500-2 (PMC4112050; doi:10.1007/s00280-014-2500-2)
Supplement: Supplementary file 1 — Supplementary material 1 (DOC 450 kb) [file 280_2014_2500_MOESM1_ESM.doc]

**Population Pharmacokinetics of Trastuzumab Emtansine (T-DM1), a HER2-Targeted Antibody–Drug Conjugate, in Patients With HER2-Positive Metastatic Breast Cancer: Clinical Implications of the Effect of Covariates**

***Cancer Chemotherapy and Pharmacology***

D. Lu,^1*^ S. Girish,^1^Y. Gao,^2^ B. Wang,^1^ J.-H. Yi,^3^ E. Guardino,^1^ M. Samant,^4^ M. Cobleigh,^5^ M. Rimawi,^6^ P. Conte,^7^ J. Y. Jin^1^

^1^Department of Clinical Pharmacology, Genentech, Inc., 1 DNA Way, South San Francisco, CA, 94080, USA. ^2^Drug Development Consulting Services, Quantitative Solutions, 845 Oak Grove Ave, Menlo Park, CA, 94025, USA. ^3^BioAnalytical Sciences, Genentech, Inc., 1 DNA Way, South San Francisco, CA, 94080, USA. ^4^Biostatistics, Genentech, Inc., 1 DNA Way, South San Francisco, CA, 94080,USA. ^5^Department of Internal Medicine, Rush University Medical Center, 1653 W. Congress Parkway, Chicago, IL, 60612, USA. ^6^Lester and Sue Smith Breast Center, Baylor College of Medicine, 6620 Main St., Houston, TX, 77030, USA. ^7^Department of Surgery, Oncology and Gastroenterology, University of Padua, via Gattamelata 64, 35128 Padua, Italy.

^*^Corresponding author: D. Lu, Department of Clinical Pharmacology, Genentech, Inc., 1 DNA Way, South San Francisco, CA, 94080, USA, e-mail: danlu@gene.com

**Supplemental Table 1.** Summary of T-DM1 studies used for the development and validation of the PopPK model

| **Parameter** | **TDM3569g**  [S1,S2] | **TDM4258g** [S3] | **TDM4374g** [S4] | **TDM4450g** [S5] | **TDM4370g (EMILIA)** [S6] | **TDM4688g** [S7] |
| --- | --- | --- | --- | --- | --- | --- |
| Phase | I | II | II | II | III | II |
| Description | Dose-escalation study in patients with HER2-positive MBC that had progressed on prior trastuzumab | Single-arm study in patients with HER2-positive MBC that had progressed on prior anti-HER2 therapy | Single-arm study in patients with HER2-positive MBC who had previously received an anthracycline, a taxane, capecitabine, lapatinib, and trastuzumab | Randomized trial of single-agent T-DM1 versus trastuzumab plus docetaxel as first-line treatment for patients with HER2-positive MBC | Randomized trial of single-agent T-DM1 versus lapatinib plus capecitabine in patients with HER2-positive LABC or MBC previously treated with a taxane and trastuzumab | Single-arm study in patients with HER2-positive MBC that had progressed on prior trastuzumab |
| T-DM1 dose | 0.3–4.8 mg/kg q3w  1.2–2.9 mg/kg qw | 3.6 mg/kg q3w | 3.6 mg/kg q3w | 3.6 mg/kg q3w | 3.6 mg/kg q3w | 3.6 mg/kg q3w |
| Patients, *n*^a^ | 52 | 109 | 110 | 66 | 334 | 51 |
| PK sampling scheme | q3w: cycle 1: days 1,^b^ 2, 3, 4/5, 8, 11, 15, and 18/19  Cycles ≥2 : day 1^c^;  qw: cycle 1: days 1,^b^ 2, 3, 4/5, 8,^c^ 11, 15,^c^ and 18/19; cycles ≥2: day 1,^c^ 8,^c^ and 15^c^ | Cycles 1^d^ and 4^d^: days 1, 8, 15,^e^ and 22^f^  Cycle 2, 3, ≥5^d^: day 1 | Cycles 1 and 4^g^: days 1, 8, and 15  Cycles 2, 3, 6, 8, 10, 12, 14, and 16^f^: day 1 | PK samples collected from patients receiving T-DM1  Cycle 1 and 5^h^: day 1, 8, 15;  Cycle 3, ≥7^h^: day 1 | PK samples collected from patients receiving T-DM1  Cycle 1 and 4^i^: day 1, 8, 15  Cycle 2, 3, 6, 8, 12, 16^i^: day 1 | Cycles 1 and 3^j^: days 1, 8, and 15  Cycles 2 and 4^j^: day 1 |

^a^Number of patients included in the PopPK analysis. All T-DM1–treated patients had scheduled PK sampling, except for those in study TDM4370g, in which only a subset of T-DM1–treated patients had scheduled PK sampling.

^b^Predose, 30 min (±15 min), and 4 h (±15 min) postinfusion on cycle 1 day 1.

^c^Predose and 30 min (±15 min) postinfusion.

^d^Predose and 30 min postinfusion samples were collected on day 1 of the cycle.

^e^Day 15 sample collection was for patients on a 21-day cycle.

^f^Day 22 sample collection was for patients on a 28-day cycle.

^g^Predose and 30 min (±10 min) postinfusion samples were collected on day 1.

^h^Predose and 30 min (±10 min) postinfusion on day 1 of every other treatment cycle.

^i^Predose and 30 min (±10 min) postinfusion on day 1 of the cycle.

^j^Predose, within 15 min postinfusion, and 60 min (±15 min) postinfusion on day 1 of cycles 1 and 3. In cycles 2 and 4, samples were taken predose only.

*HER2* human epidermal growth factor receptor 2, *LABC* locally advanced breast cancer, *MBC* metastatic breast cancer, *PK* pharmacokinetic, *PopPK* population pharmacokinetic, *q3w* every 3 weeks, *qw* weekly, *T-DM1* trastuzumab emtansine

**Supplemental Table 2.** Covariates tested in the PopPK analysis

|  | | **Covariates tested** |
| --- | --- | --- |
| **Demographics** | | Age at baseline  Body weight  Body mass index^a^  Body surface area^b^  Race (white, Asian, other)  Region (Asia, non-Asia) |
| **Covariates related to disease severity and treatment history** | **Tumor burden** | Baseline sum of longest dimension of target lesions  Number of nontarget lesions  Disease measurability (yes/no) |
|  | **Metastasis status** | Number of metastatic sites at baseline  Bone metastasis (yes/no)  Liver metastasis (yes/no)  Lung metastasis (yes/no)  Visceral disease (yes/no) |
|  | **HER2 expression** | Baseline serum HER2 shed extracellular domain concentration  HER2-positive centrally confirmed status (yes/no) |
|  | **Health status** | Baseline Eastern Cooperative Oncology Group performance status score |
|  | **Treatment history** | Baseline trastuzumab concentration  Prior systemic therapy in locally advanced/metastatic setting (yes/no) |
| **Covariates related to renal function** | | Baseline creatinine clearance  Baseline serum creatinine concentration |

*HER2* human epidermal growth factor receptor 2, *PopPK* population pharmacokinetic ^a^Defined by the Quetelet index [S8]: [(body weight)/(height^2)]
^b^Defined by the DuBois and DuBois formula [S9]: [(0.007184) × (body weight^0.425) × (height^0.725)].

**Supplemental Table 3 Baseline population characteristics for all patients by study in the PopPK model development dataset and the model validation dataset**

| **Characteristics** | **Model development dataset:  data from TDM3569g, TDM4258g, TDM4374g, TDM4450g, and TDM4370g (EMILIA)** | | | | **Model validation dataset: data from TDM4688g** | | |
| --- | --- | --- | --- | --- | --- | --- | --- |
| Patients, *n*^a^ | 671 | | | | 51 | | |
| PK samples, *n* | 9934 | | | | 505 | | |
| **Continuous covariates** | | | | | | | |
| **Covariates** | ***N*** | | **Median**  **[min, max]** | | ***N*** | **Median**  **[min, max]** | |
| Age (y) | 671 | | 53.0  [27.0, 84.0] | | 51 | 52.0  [34.0, 85.0] | |
| Body weight (kg) | 670 | | 68.0  [37.7, 137.4] | | 51 | 67.2  [44.0, 105.3] | |
| CrCL (mL/min) | 669 | | 92.9  [25.6, 294.6] | | 51 | 101.3  [53.1, 170] | |
| ALBU (g/L) | 660 | | 41.0  [22.0, 57.0] | | 51 | 40.0  [31.0, 47.0] | |
| TPRO (g/L) | 660 | | 71.0  [54.0, 89.0] | | 51 | 71.0  [58.0, 83.0] | |
| TBIL (µmol/L) | 667 | | 7.0  [1.7, 59.0] | | 51 | 5.1  [1.7, 22.2] | |
| AST (U/L) | 667 | | 27.0  [4.0, 226.5] | | 51 | 29.0  [13.0, 235.0] | |
| ALT (U/L) | 665 | | 24.0  [5.0, 173.0] | | 51 | 22.0  [6.0, 179.0] | |
| ALKP (U/L) | 667 | | 86.0  [29.5, 652.0] | | 51 | 95.0  [40.0, 338.0] | |
| ECD (ng/mL) | 639 | | 25.2  [5.0, 3500.0] | | 50 | 25.8  [7.1, 1420.0] | |
| TBL (µg/mL) | 671 | | 0.5  [0.0, 124.0] | | 51 | 1.9  [0.0, 148.0] | |
| TMBD (cm) | 585 | | 8.6  [1.0, 52.0] | | 43 | 5.10  [1.15, 18.20] | |
| INR | 649 | | 1  [0.74, 3.3] | | NA | NA | |
| APTT (sec) | 643 | | 29  [8.2, 79] | | NA | NA | |
| **Categorical covariates** | | | | | | | |
| **Covariates** | ***N*** | **% of total** | | ***N*** | | | **% of total** |
| Sex (F/M) | 667/4 | 99.4/0.6 | | 51/0 | | | 100/0 |
| Race (Asian/Other/White) | 73/57/541 | 10.9/8.5/80.6 | | 2/5/44 | | | 3.9/9.8/86.3 |
| Region (Asia/Western Europe/Other/United States) | 58/145/66/402 | 8.6/21.6/9.8/59.9 | | 0/0/0/51 | | | 0/0/0/100 |
| MSCT (<3 or ≥3) | 303/368 | 45.2/54.8 | | 10/41 | | | 19.6/80.4 |
| ECOG performance status score (0/1/2/3) | 401/259/10/1 | 59.8/38.6/1.5/0.1 | | 28/23/0/0 | | | 54.9/45.1/0/0 |
| NNT (0–1 or 2+) | 211/460 | 31.4/68.6 | | 17/34 | | | 33.3/66.7 |
| BMT (Yes/No/Missing) | 306/355/10 | 45.6/52.9/1.5 | | 35/16/0 | | | 68.6/31.4/0 |
| HER2+ (Yes/No/Missing) | 567/51/53 | 84.5/7.6/7.9 | | 38/5/8 | | | 74.5/9.8/15.7 |
| LIMT (Yes/No/Missing) | 285/380/6 | 42.5/56.6/0.9 | | 29/22/0 | | | 56.9/43.1/0 |
| LUMT (Yes/No/Missing) | 327/336/8 | 48.7/50.1/1.2 | | 34/17/0 | | | 66.7/33.3/0 |
| DME (Yes/No) | 586/85 | 87.3/12.7 | | 43/8 | | | 84.3/15.7 |
| PST (Yes/No) | 568/103 | 84.6/15.4 | | 48/3 | | | 94.1/5.9 |
| VISC (Yes/No) | 475/196 | 70.8/29.2 | | 42/9 | | | 82.4/17.6 |

^a^Number of patients included in the PopPK analysis.

*ALBU* serum albumin concentration, *ALKP* serum alkaline phosphatase concentration, *ALT* serum alanine aminotransferase concentration, *APTT* activated partial thromboplastin time, *AST* serum aspartate aminotransferase concentration, *BMT* bone metastasis, *CrCL* baseline creatinine clearance, *DME* disease measurability, *ECD* baseline serum human epidermal growth factor receptor 2 shed extracellular domain concentration, *ECOG* Eastern Cooperative Oncology Group, *F* female, *HER2+* human epidermal growth factor receptor 2–positive (centrally confirmed) status, *INR* international normalized ratio, *LIMT* liver metastasis, *LUMT* lung metastasis, *M* male, *max* maximum, *min* minimum, *MSCT* baseline metastatic site count, *NA* not available, *NNT* number of nontargeted lesions, *PK* pharmacokinetic, *PopPK* population pharmacokinetic, *PST* prior systemic therapy in the locally advanced/metastatic setting, *TBIL* total bilirubin, *TBL* baseline trastuzumab concentration, *TMBD* baseline sum of longest dimension of target lesions, *TPRO* total protein, *VISC* visceral disease

Missing covariates were treated as follows:

Covariates missing for ≤15% of patients: continuous covariates were imputed as the population median, and categorical covariates were imputed as the most frequent category. Covariates missing for >15% of the patients: no imputation.

**Supplemental Table 4. Impact of age, race, region, and renal function on steady-state T-DM1 exposure after repeated dosing of 3.6 mg/kg by 0.5-h IV infusion q3w**

| **Characteristics** | **Age** | | | **Race** | | **Region** | | | | **Renal function** | | | |
| --- | --- | --- | --- | --- | --- | --- | --- | --- | --- | --- | --- | --- | --- |
|  | **<65 years** | **65−75 years** | **>75 years** | **Non-Asian** | **Asian** | **US** | **Western Europe** | **Asia** | **Other** | **Normal** | **Mild impairment** | **Moderate impairment** | **Severe impairment** |
| No. of patients (%) | 577 (85.99) | 78 (11.62) | 16  (2.39) | 598 (89.12) | 73 (10.88) | 402 (59.91) | 145 (21.61) | 58  (8.64) | 66  (9.84) | 361  (53.96) | 254  (37.97) | 53  (7.92) | 1  (0.15) |
| Mean AUC, day*µg/mL (5th–95th percentile) | 367.2 (247.8, 514.0) | 367.0 (249.5, 483.4) | 370.2 (251.2, 547.0) | 369.8 (249.7, 517.2) | 346.1 (245.9, 427.8) | 365.8 (241.6, 529.2) | 375.2 (269.0, 483.7) | 351.0 (254.0, 427.1) | 372.6  (258.3, 472.6) | 391.6  (264.7, 524.8) | 360.1  (218.0, 485.5) | 350.2  (217.5, 493.3) | 375.5 |
| Mean C_max_, µg/mL (5th–95th percentile) | 80.67 (67.63, 99.47) | 81.47 (66.50, 98.22) | 80.36 (64.06, 97.32) | 81.03 (66.40, 100.8) | 78.53 (67.59, 88.39) | 79.81 (65.12, 101.33) | 83.79 (71.08, 97.81) | 78.11 (67.30, 88.61) | 82.15  (70.49, 93.97) | 85.45  (71.33, 102.7) | 79.60  (65.31, 94.99) | 77.65  (62.09, 96.09) | 78.08 |
| Mean C_trough_, µg/mL (5th–95th percentile) | 2.277 (0.447, 4.699) | 2.225 (0.374, 4.253) | 3.119 (0.554, 7.940) | 2.340 (0.464, 4.813) | 1.892 (0.328, 3.361) | 2.376 (0.404, 5.034) | 2.201 (0.530, 4.002) | 1.982 (0.639, 3.452) | 2.244  (0.414, 4.194) | 2.390  (0.507, 4.755) | 2.295  (0.408, 4.607) | 2.547  (0.285, 4.708) | 3.161 |

*AUC* area under the serum concentration versus time curve, *C_max_* maximum concentration, *C_trough_* trough concentration, *IV* intravenous, *q3w* every 3 weeks, *T-DM1* trastuzumab emtansine, *US* United States

**Supplemental Fig. 1** T-DM1 PopPK model diagram

*CL* elimination clearance, *D_i_* (*i*=1….*n*) *ith* dose at time *tDi*, *k_10_* first-order elimination rate constant, *k_12_* first-order rate constant from central to peripheral, *k_21_* first-order rate constant from peripheral to central, *PopPK* population pharmacokinetic, *Q* distribution clearance, *R_0_* zero-order drug infusion rate, *t* sample time, *tD_i_* (*i*=1….*n*) time of the *ith* dose, *T_inf_* infusion duration, *T-DM1* trastuzumab emtansine, *V_c_* volume of distribution in the central compartment, *V_p_* volume of distribution in the peripheral compartment, *X_1_* drug amount in the central compartment, *X_2_* drug amount in the peripheral compartment

**Supplemental Fig. 2** Predicted versus observed goodness-of-fit plots for the final PopPK model

IPRED versus observed T-DM1 concentrations (left) and PRED versus observed serum T-DM1 concentrations (right) for the final PopPK model on a logarithmic scale. Points are individual data, and lines represent the unit diagonal. IPRED individual predicted serum T-DM1 concentrations, PopPK population pharmacokinetic, PRED population-predicted serum T-DM1 concentration, T-DM1 trastuzumab emtansine

**Supplemental Fig. 3** Residual goodness-of-fit plots for the final PopPK model

The residual plots of serum T-DM1 concentrations for the final PopPK model. IWRES versus time (upper left), CWRES versus time (upper right), IWRES versus PRED (lower left), CWRES versus PRED (lower right). Points are individual data. Red solid lines represent the unit line at zero. Blue solid lines represent the absolute value of CWRES of six. CWRES conditional weighted residuals, IWRES individual weighted residuals, PopPK population pharmacokinetic, PRED population-predicted serum T-DM1 concentrations, T-DM1 trastuzumab emtansine

**Supplemental Fig. 4** VPC of T-DM1 serum concentration–time profiles for the 3.6-mg q3w subset in the (a) model development dataset and (b) model validation dataset (TDM4866g, 3.6 mg/kg, n=51) [S7]

Points are the observed T-DM1 serum concentrations. The red lines are the median of the predicted concentrations by the final PopPK model (1,000 trials). The blue shaded areas are the spread (5th to 95th percentile) of the predicted concentrations. Vertical dashed lines indicate the time of dosing. *PopPK* population pharmacokinetic, *q3w* every 3 weeks, *T-DM1* trastuzumab emtansine, *VPC* visual predictive check

**Supplemental Fig. 1**

**
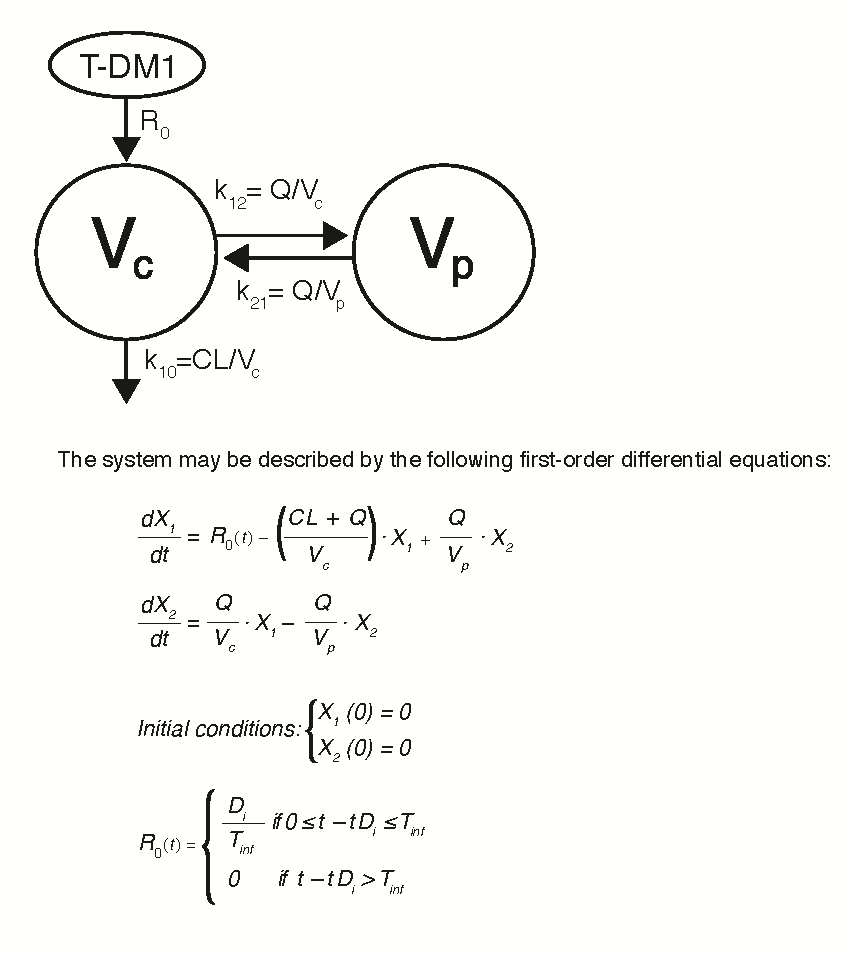
**

**Supplemental Fig. 2**


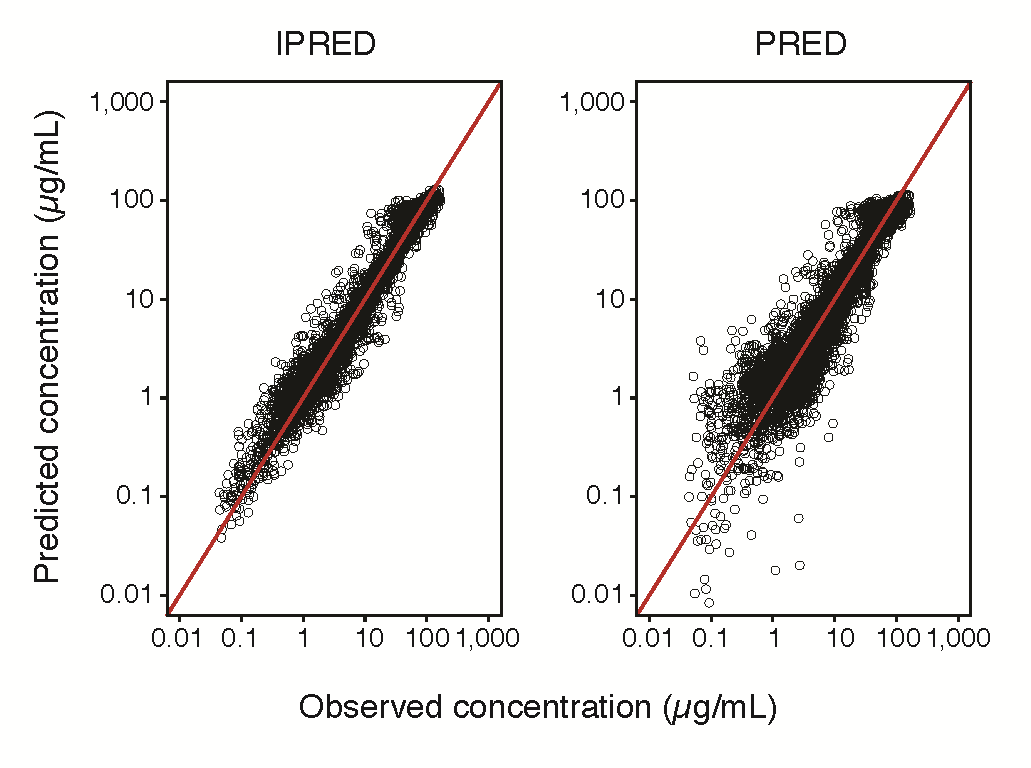


**Supplemental Fig. 3**


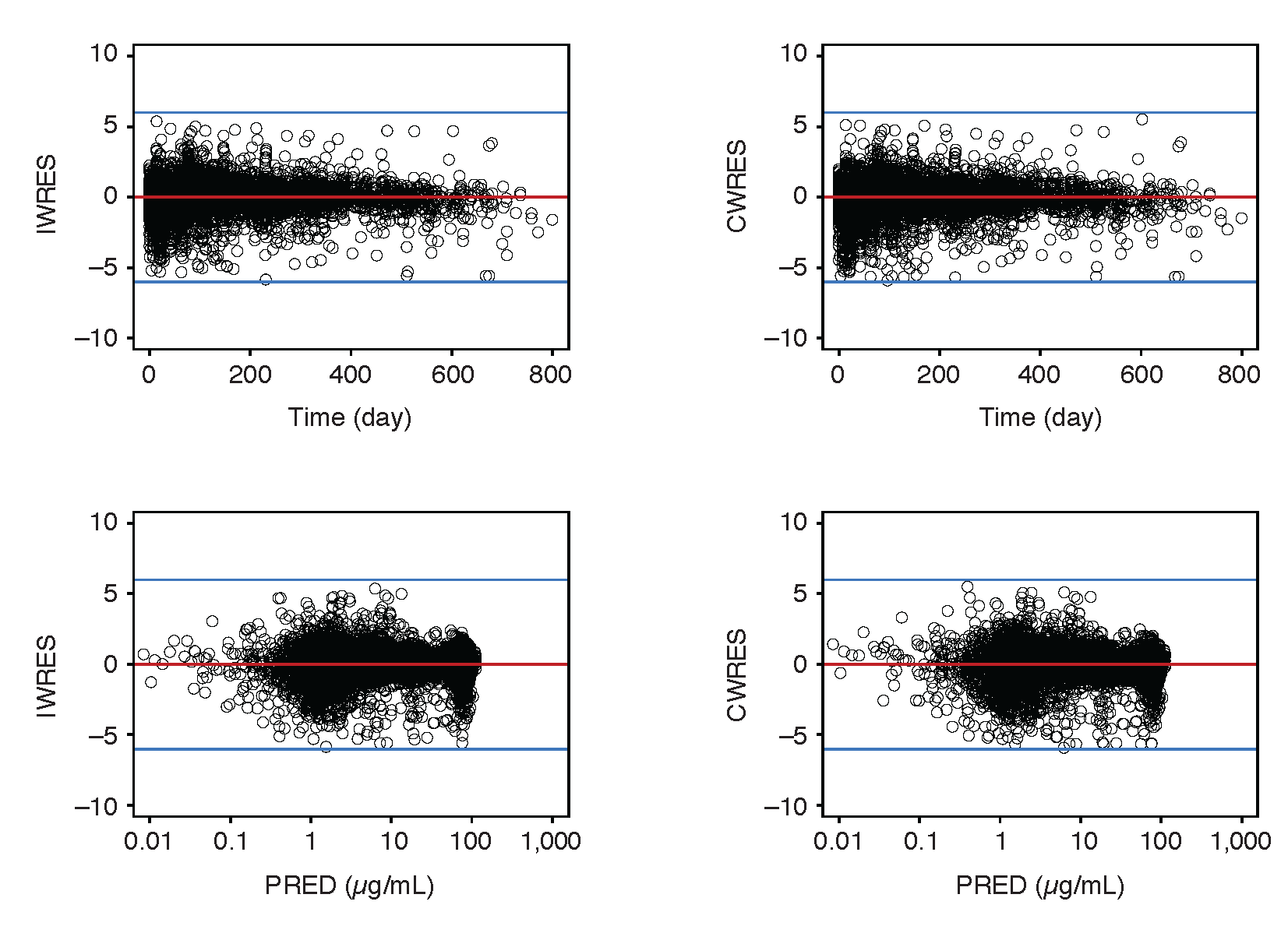


**Supplemental Fig. 4.**


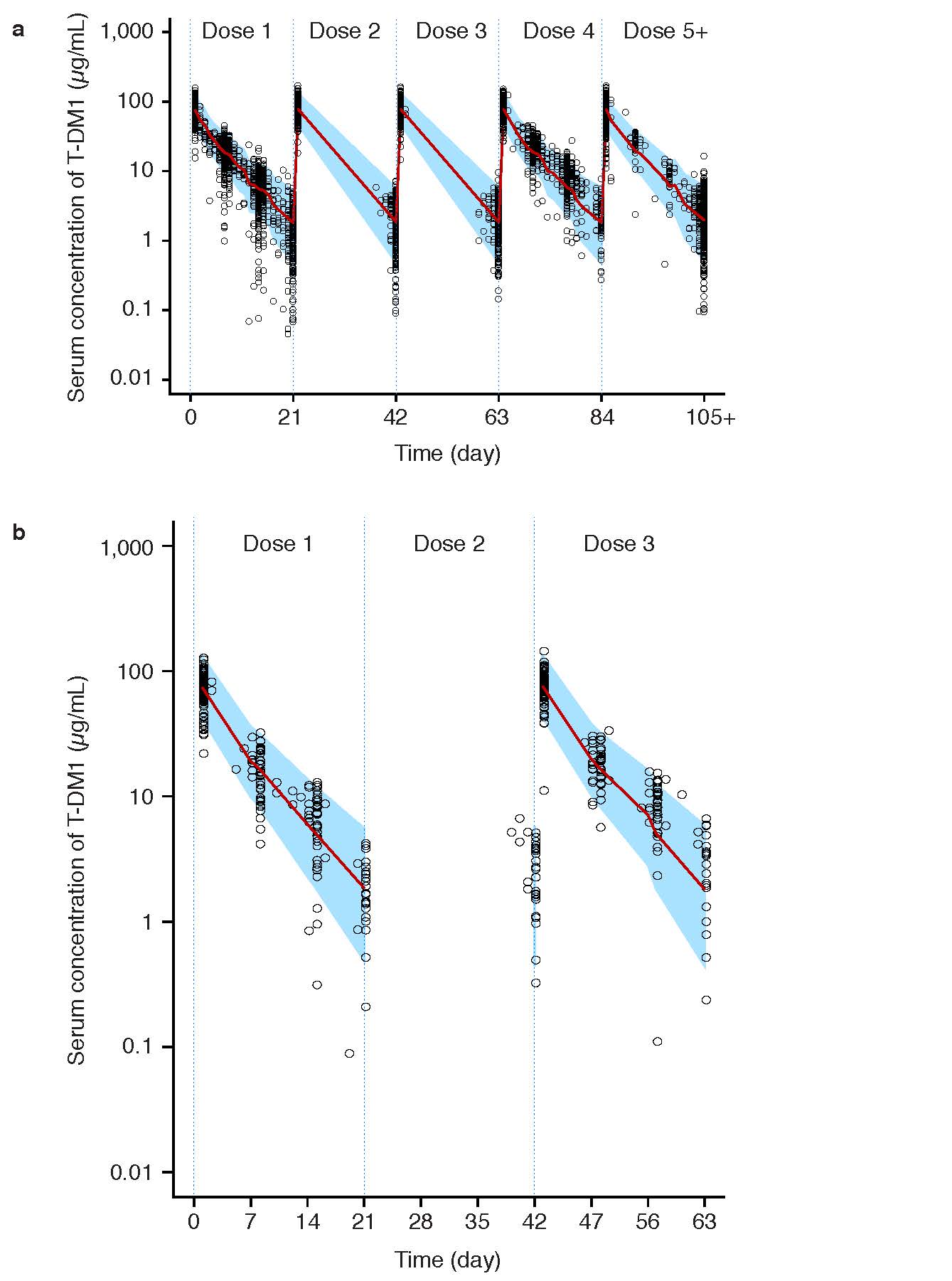


**Supplemental References**

S1. Krop IE, Beeram M, Modi S, Jones SF, Holden SN, Yu W, [Girish S](http://www.ncbi.nlm.nih.gov/pubmed?term=Girish S%5BAuthor%5D&cauthor=true&cauthor_uid=20421541), [Tibbitts J](http://www.ncbi.nlm.nih.gov/pubmed?term=Tibbitts J%5BAuthor%5D&cauthor=true&cauthor_uid=20421541), [Yi JH](http://www.ncbi.nlm.nih.gov/pubmed?term=Yi JH%5BAuthor%5D&cauthor=true&cauthor_uid=20421541), [Sliwkowski MX](http://www.ncbi.nlm.nih.gov/pubmed?term=Sliwkowski MX%5BAuthor%5D&cauthor=true&cauthor_uid=20421541), [Jacobson F](http://www.ncbi.nlm.nih.gov/pubmed?term=Jacobson F%5BAuthor%5D&cauthor=true&cauthor_uid=20421541), [Lutzker SG](http://www.ncbi.nlm.nih.gov/pubmed?term=Lutzker SG%5BAuthor%5D&cauthor=true&cauthor_uid=20421541), [Burris HA](http://www.ncbi.nlm.nih.gov/pubmed?term=Burris HA%5BAuthor%5D&cauthor=true&cauthor_uid=20421541) (2010) Phase I study of trastuzumab-DM1, an HER2 antibody-drug conjugate, given every 3 weeks to patients with HER2-positive metastatic breast cancer. J Clin Oncol 28:2698–2704

S2. Beeram M, Krop IE, Burris HA, Girish SR, Yu W, Lu MW, Holden SN, Modi S (2012) A phase 1 study of weekly dosing of trastuzumab emtansine (T-DM1) in patients with advanced human epidermal growth factor 2-positive breast cancer. Cancer 118:5733–5740

S3. Burris HA 3rd, Rugo HS, Vukelja SJ, Vogel CL, Borson RA, Limentani S, [Tan-Chiu E](http://www.ncbi.nlm.nih.gov/pubmed?term=Tan-Chiu E%5BAuthor%5D&cauthor=true&cauthor_uid=21172893), [Krop IE](http://www.ncbi.nlm.nih.gov/pubmed?term=Krop IE%5BAuthor%5D&cauthor=true&cauthor_uid=21172893), [Michaelson RA](http://www.ncbi.nlm.nih.gov/pubmed?term=Michaelson RA%5BAuthor%5D&cauthor=true&cauthor_uid=21172893), [Girish S](http://www.ncbi.nlm.nih.gov/pubmed?term=Girish S%5BAuthor%5D&cauthor=true&cauthor_uid=21172893), [Amler L](http://www.ncbi.nlm.nih.gov/pubmed?term=Amler L%5BAuthor%5D&cauthor=true&cauthor_uid=21172893), [Zheng M](http://www.ncbi.nlm.nih.gov/pubmed?term=Zheng M%5BAuthor%5D&cauthor=true&cauthor_uid=21172893), [Chu YW](http://www.ncbi.nlm.nih.gov/pubmed?term=Chu YW%5BAuthor%5D&cauthor=true&cauthor_uid=21172893), [Klencke B](http://www.ncbi.nlm.nih.gov/pubmed?term=Klencke B%5BAuthor%5D&cauthor=true&cauthor_uid=21172893), [O'Shaughnessy JA](http://www.ncbi.nlm.nih.gov/pubmed?term=O'Shaughnessy JA%5BAuthor%5D&cauthor=true&cauthor_uid=21172893) (2011) Phase II study of the antibody drug conjugate trastuzumab-DM1 for the treatment of human epidermal growth factor receptor 2 (HER2)-positive breast cancer after prior HER2-directed therapy. J Clin Oncol 29:398–405

S4. Krop IE, LoRusso P, Miller KD, Modi S, Yardley D, Rodriguez G, [Guardino E](http://www.ncbi.nlm.nih.gov/pubmed?term=Guardino E%5BAuthor%5D&cauthor=true&cauthor_uid=22649126), [Lu M](http://www.ncbi.nlm.nih.gov/pubmed?term=Lu M%5BAuthor%5D&cauthor=true&cauthor_uid=22649126), [Zheng M](http://www.ncbi.nlm.nih.gov/pubmed?term=Zheng M%5BAuthor%5D&cauthor=true&cauthor_uid=22649126), [Girish S](http://www.ncbi.nlm.nih.gov/pubmed?term=Girish S%5BAuthor%5D&cauthor=true&cauthor_uid=22649126), [Amler L](http://www.ncbi.nlm.nih.gov/pubmed?term=Amler L%5BAuthor%5D&cauthor=true&cauthor_uid=22649126), [Winer EP](http://www.ncbi.nlm.nih.gov/pubmed?term=Winer EP%5BAuthor%5D&cauthor=true&cauthor_uid=22649126), [Rugo HS](http://www.ncbi.nlm.nih.gov/pubmed?term=Rugo HS%5BAuthor%5D&cauthor=true&cauthor_uid=22649126) (2012) A phase II study of trastuzumab emtansine in patients with human epidermal growth factor receptor 2-positive metastatic breast cancer who were previously treated with trastuzumab, lapatinib, an anthracycline, a taxane, and capecitabine. J Clin Oncol 30:3234–3241

S5. Hurvitz SA, Dirix L, Kocsis J, Bianchi GV, Lu J, Vinholes J, [Guardino E](http://www.ncbi.nlm.nih.gov/pubmed?term=Guardino E%5BAuthor%5D&cauthor=true&cauthor_uid=23382472), [Song C](http://www.ncbi.nlm.nih.gov/pubmed?term=Song C%5BAuthor%5D&cauthor=true&cauthor_uid=23382472), [Tong B](http://www.ncbi.nlm.nih.gov/pubmed?term=Tong B%5BAuthor%5D&cauthor=true&cauthor_uid=23382472), [Ng V](http://www.ncbi.nlm.nih.gov/pubmed?term=Ng V%5BAuthor%5D&cauthor=true&cauthor_uid=23382472), [Chu YW](http://www.ncbi.nlm.nih.gov/pubmed?term=Chu YW%5BAuthor%5D&cauthor=true&cauthor_uid=23382472), [Perez EA](http://www.ncbi.nlm.nih.gov/pubmed?term=Perez EA%5BAuthor%5D&cauthor=true&cauthor_uid=23382472) (2013) Phase II randomized study of trastuzumab emtansine versus trastuzumab plus docetaxel in patients with human epidermal growth factor receptor 2-positive metastatic breast cancer. J Clin Oncol 31:1157–1163. Erratum in J Clin Oncol 2013;31:2977

S6. Verma S, Miles D, Gianni L, Krop IE, Welslau M, Baselga J, [Pegram M](http://www.ncbi.nlm.nih.gov/pubmed?term=Pegram M%5BAuthor%5D&cauthor=true&cauthor_uid=23020162), [Oh DY](http://www.ncbi.nlm.nih.gov/pubmed?term=Oh DY%5BAuthor%5D&cauthor=true&cauthor_uid=23020162), [Diéras V](http://www.ncbi.nlm.nih.gov/pubmed?term=Diéras V%5BAuthor%5D&cauthor=true&cauthor_uid=23020162), [Guardino E](http://www.ncbi.nlm.nih.gov/pubmed?term=Guardino E%5BAuthor%5D&cauthor=true&cauthor_uid=23020162), [Fang L](http://www.ncbi.nlm.nih.gov/pubmed?term=Fang L%5BAuthor%5D&cauthor=true&cauthor_uid=23020162), [Lu MW](http://www.ncbi.nlm.nih.gov/pubmed?term=Lu MW%5BAuthor%5D&cauthor=true&cauthor_uid=23020162), [Olsen S](http://www.ncbi.nlm.nih.gov/pubmed?term=Olsen S%5BAuthor%5D&cauthor=true&cauthor_uid=23020162), [Blackwell K](http://www.ncbi.nlm.nih.gov/pubmed?term=Blackwell K%5BAuthor%5D&cauthor=true&cauthor_uid=23020162); [EMILIA Study Group](http://www.ncbi.nlm.nih.gov/pubmed?term=EMILIA Study Group%5BCorporate Author%5D) (2012) Trastuzumab emtansine for HER2-positive advanced breast cancer. N Engl J Med 367:1783–1791. Erratum in N Engl J Med 2013;368:2442

S7. Gupta M, Wang B, Carrothers TJ, LoRusso PM, Chu Y-W, Shih T, Loecke D, Joshi A, Saad O, Yi J-H, Girish S (2013) Effects of trastuzumab emtansine (T-DM1) on QT interval and safety of pertuzumab plus T-DM1 in patients with previously treated human epidermal growth factor receptor 2–positive metastatic breast cancer. Clin Pharmacol Drug Devel 2:11–24

S8. Keys A, Fidanza F, Karvonen MJ, Kimura N, Taylor HL (1972) Indices of relative weight and obesity. J Chronic Dis 25:329–343

# S9. Du Bois D, Du Bois EF (1989) A formula to estimate the approximate surface area if height and weight be known. 1916. Nutrition 5:303–311; discussion 312–313
